# Robust Estimation of Breast Cancer Incidence Risk in Presence of Incomplete or Inaccurate Information

**DOI:** 10.31557/APJCP.2020.21.8.2307

**Published:** 2020-08

**Authors:** Siva Teja Kakileti, Geetha Manjunath, Andre Dekker, Leonard Wee

**Affiliations:** 1 *Niramai Health Analytix Pvt Ltd., Koramangala, Bangalore, Karnataka, India. *; 2 *Department of Radiation Oncology (MAASTRO Clinic), GROW School for Oncology and Developmental Biology, Maastricht University Medical Centre+, Maastricht, The Netherlands. *

**Keywords:** Breast cancer risk, machine learning, artificial neural networks, missing values, inaccurate data

## Abstract

**Purpose:**

To evaluate the robustness of multiple machine learning classifiers for breast cancer risk estimation in the presence of incomplete or inaccurate information.

**Data and methods:**

Open data for this study was obtained from the BCSC Data Resource (http://breastscreening.cancer.gov/). We conducted two ablation-type experiments to compare the robustness of different classifiers where we randomly switched known information to missing with a missing probability of p_m_ in one experiment, and randomly corrupted the existing information with a probability of p_c_ in another experiment. We considered three prominent machine-learning classifiers such as Logistic regression (LR), Random Forests (RF) and a custom Neural Network (NN) architecture and compared their degradation of discrimination performance as a function of increasing probability of missing or inaccurate data.

**Results:**

LR, RF and custom NN resulted in an Area Under Curve (AUC) of 0.645, 0.643 and 0.649, respectively, on a test set with 500,000 total observations. When we manipulated the data by varying probabilities p_m_ and p_c_ from 0 to 1, NN resulted in better performance in terms of AUC compared to RF and LR as long as less than half the data was missing/inaccurate (that is, for values of p_m_ < 0.5 and p_c_ < 0.5). However, for missing (p_m_) or corruption (p_c_) probabilities above 0.5, LR gave similar performance as the custom NN. RF resulted in overall poorer performance when the data had additional missing or incorrect entries.

**Conclusion:**

In cases where the input information is missing or inaccurate, our experiments show that the proposed custom NN provides reliable risk estimates in medical datasets like BCSC. These results are particularly important in health care applications where not every attribute of the individual participant might be available.

## Introduction

The World Health Organization estimates that 1 in 12 women are diagnosed with some breast abnormality in their lifetime (Ferlay et al., 2015; Fitzmaurice et al., 2015). In developing countries like India, mortality rates approach 50% (Bray et al., 2013; Ferlay et al., 2015; Malvia et al., 2017). The national incidence and mortality rates of breast cancer in India are approximately 25.8 and 12.7 per 100,000 women, respectively (Bray et al., 2013; Ferlay et al., 2015; Fitzmaurice et al., 2015; Malvia et al., 2017). This incidence rises to 35-40 per 100,000 women in large metropolitan cities (such as Delhi, Chennai and Bangalore) (Malvia et al., 2017). There is a critical imbalance of 1 radiologist per 100,000 persons across India, suggesting that access to appropriate expertise and screening facilities are major factors impacting detrimentally on breast cancer mortality (Kalyanpur, 2008). 

There is low public awareness and limited acceptability of breast cancer screening among Indian women. Significant efforts are needed to improve early breast cancer detection facilities for improving access (Bagacchi, 2016; Kalyanpur, 2008; Ragavan, 2008). It has been found that risk-based population screening is more effective than unselective population screening. The use of multifactorial mathematical models to predict individual risk may help to promote self-awareness and equip individuals with the necessary information to address their situation. Previous studies show that an individual’s absolute risk is modulated by known health and environmental factors (Amir et al., 2003; Barlow et al., 2006; Claus et al, 1991; Colditz et al., 1996; Dupont and Page, 1985; Evans and Howell, 2007; Ford et al., 1998; Gail et al., 1989; Hartmann et al., 2005; McPherson et al., 2000), presence of any BRCA genetic mutation (Claus et al, 1991; Ford et al., 1998; Thompson et al., 2004) and having a family history of breast cancer (Claus et al, 1991; Colditz et al., 1996; McPherson et al., 2000). 

Among the prominent risk models, the Gail model (Gail et al., 1989) assesses risk based on a participant’s current age, age at menarche, age at first childbirth, race, ethnicity, number of first-degree relatives with a history of breast cancer and the number of breast biopsy examinations. This risk model was based on the data collected from the Breast Cancer Detection Demonstration Project (BCDDP) in 1980. The Claus model (Claus et al., 1991) derives the risk score from age and detailed family cancer history involving the number of first- and second-degree relatives and the age of onset of breast cancer in a relative. Amir et al. (Amir et al., 2003) used age, body mass index, age at first childbirth, age at menarche, menopausal status, the number of first- and second-degree relatives with cancer, age of onset of breast cancer in a relative, bilateral cancer in a relative, ovarian cancer in a relative, hormonal exposure and benign breast disease history to predict risk. Recently, Barlow et al. (Barlow et al., 2006) used data collected through the Breast Cancer Surveillance Consortium (BCSC) (BCSC, 2018) to assess risk using a comprehensive list of risk factors, as shown in Table 1.

However, the above widely used risk prediction models in literature are no longer usable if any of the required parameters are missing or incorrect. Complete medical histories may not always be available in the real world; especially in developing countries, individuals often choose not to disclose details due to fear of social stigmatization, or they may report incorrect information due to a flawed recollection. A predictive classifier algorithm intended to be used for risk estimation in this setting should, therefore, be tested for robustness with respect to either missing and/or incorrect data. Our focus in this investigation was to compare the robustness of risk prediction models under the constraint of limited or incorrect information.

In a recent paper, Deist et al., (2018) compared various machine learning (ML) classifiers for medical situations such as lung cancer, head and neck cancer, laryngeal cancer and meningioma detection. They observed that relatively simple approaches such as logistic regression and random forests resulted in broadly better discriminatory performance if complete and accurate information was available. However, this analysis was only applied to complete cases with no missing values and assumed no corrupted values.

In this paper, we evaluated the robustness of multiple machine learning-based classifier algorithms for risk prediction in the presence of incomplete and inaccurate information. For this, we added simulated incomplete and inaccurate information into a large medical dataset and measured the degradation of discrimination performance. Section 2 of this paper discusses the dataset and our proposed methodology, followed by results in section 3. In section 4, we discussed the comparative performance of the machine learning classifiers. We also considered a visualization of results for better clinical interpretation of the predicted risk scores.

## Materials and Methods

*Dataset*

The data for this study was obtained from the BCSC Data Resource (more information at http://breastscreening.cancer.gov/). The BCSC (BCSC, 2018) dataset aggregated risk factors of women who attended breast cancer screening from 1st January 1996 to 31st December 2002 from seven data registries covering most mammography clinic locations in the United States. The detailed information of 2.4 million observations, of women aged between 35 and 85 years, attending a mammography clinic was collected by a questionnaire for the BCSC study. Specific exclusions were: women with prior breast cancer or breast augmentation, or women who had already undergone breast cancer screening in the preceding 12 months. The primary endpoint was diagnostic confirmation of either ductal carcinoma in situ or invasive breast carcinoma after one year. Overall, the participation rate was highest in the 50-54 age group as shown in Table 1. Women from a diverse range of ethnic backgrounds were included. Approximately 20% of values were initially missing in the BCSC dataset. These missing values were due to incomplete or withheld responses on the survey questionnaires. 

The BCSC dataset recorded: menopausal status, age, breast density, race, Hispanic ethnicity, Body Mass Index (BMI), age at first childbirth, number of first-degree relatives affected with breast cancer (NRBC), information about previous breast medical procedures, result from last mammogram, incidence of surgical menopause and treatment by Hormone Replacement Therapy (HRT). All data elements were coded as categorical variables.

In total, 2 392 998 rows of observations were available of 1,007,660 unique subjects that met the following criteria: (1) at least one previous mammogram within the preceding 5 years, (2) had not undergone mammography in the last one year from the time of registration and (3) had no prior history of breast cancer. Each BCSC risk factor was categorized according to the coding schema shown in Table 1. A total of 11,638 women were diagnosed with breast cancer within one year of screening, out of which 9,335 cases were invasive breast cancer and 2,303 were ductal carcinoma in situ.

*Selection of machine learning classifiers*

We selected three of the most commonly used types of ML classifiers for our analysis – namely logistic regression, random forest and deep neural networks. While this selection of ML-based classifiers is similar to a recent paper (Deist et al., 2018), our focus is on analyzing the performance of these models in the presence of additional missing elements and inaccurate data entry. 

Logistic regression (Walker and Duncan, 1967) models aim to optimize the linear relationship between the log of odds (logit) of the dependent variable (outcome) and the independent variables such that the overall error between predicted and true outcomes for different observations is minimized. 

Random forests (Ho, 1995) create a large ensemble of individually distinct decision trees, such that the final classification is derived from the majority voting result taken over all trees. In our experiments, we have considered a random forest of 200 decision trees with a maximum depth of 10 for each decision tree.

Neural Networks (NN) establish complex non-linear relationships between the inputs and the output using interconnected hidden layers. Given that we have a sufficiently large dataset, use of NN was feasible (Bishop, 1995). We experimented with a variety of NN configurations and found that a simple 3-layer NN architecture generalized well for breast cancer risk prediction on the BCSC training dataset.

The first stage of the NN architecture consisted of non-linear transformations to produce 64 different non-linear variations of each individual risk factor using (1x1) convolution filters and ReLU activations. The resulting 64 channels were then convolved with 32 (1x1) filters followed by ReLU activations to reduce the dimensionality. The resulting intra-input variations were mixed using a fully inter-connected dense layer consisting of 1,024 nodes to learn the non-linear inter-relations across different risk factors. A dropout layer (Goodfellow et al., 2016) was applied immediately after the dense layer for regularization and to avoid over-fitting. The final output layer consisted of two output nodes with soft-max activation, representing the class probabilities of getting cancer in the next year and the probability of not getting cancer in next year, respectively.

The starting weights of the NN were initialized randomly. We then used an Adam optimizer for updating NN parameters iteratively based on the training set because of its reported superior results. The Adam optimizer was set to learning rate 0.001 and a batch size of 15,000 and dropout probability of 0.5 was used for training. Categorical cross-entropy (Goodfellow et al., 2016) was used as the loss function to measure the error between predicted and true probability distributions. The output corresponding to the probability of cancer obtained from the soft-max layer was used as the absolute final risk score in our analysis. The NN was implemented with the Keras (Keras, 2018) python library and a Tensorflow back-end (Abadi et al., 2016). 

*Performance evaluation*

As mentioned in the Introduction, incomplete or inaccurate information may be encountered during real-world health data collection, which could result in inaccurate risk estimation. A good classifier needs to be robust to these errors and should result in a risk value that is close to the true risk, in spite of missing or inaccurate information. To quantify the robustness of different classifiers in this setting, we conducted the two experiments as described below.

*Experiment 1: Robustness to incomplete data*

In order to evaluate the robustness of the classifiers to missing or incomplete data, we randomly switched one of the existing known values to missing values with a probability of ‘0 < p_m_ < 1’ during validation and testing. We varied the value of ‘p_m_’ in steps of 0.1. We first trained each of the classifiers by introducing additional 10% of missing values in the training set.

*Experiment 2: Robustness to inaccurate data*

In a similar manner to Experiment 1, we randomly modified the data by replacing the feature values with a different value, with probability of ‘0 < p_c_ < 1’. As before, we varied the value of p_c_ in steps of 0.1.

To assess the degradation of discrimination performance, we used an Area Under Receiver Operating Characteristic curve (AUC) metric (Zweig and Campbell, 1993). AUC measures the probability of correctly classifying the subjects according to a binary outcome. For perfect classification, we would obtain an AUC value of 1. For a model that had no discriminatory power beyond random guessing, we would expect an AUC value of 0.5. It is generally interpreted that a higher value of AUC suggests better performance in differentiating between the two available outcomes. 

*Training and cross-validation settings*

The entire BCSC dataset was randomly divided into a model development set (75%) and a model testing set (25%). The training and validation sets had the same proportion of non-cancer and cancer findings. For cross-validation, we further split the training set in a ratio of 3:1 into training and cross-validation subsets.

## Results

We first compared the performance of all three classifiers on the original BCSC dataset. Table 2 shows the mean AUC values across 10 repeated trials for logistic regression, random forests and the custom NN. The NN resulted in slightly better performance followed by logistic regression and random forests. 

*Performance of classifiers with incomplete information*

**Figure 1:**
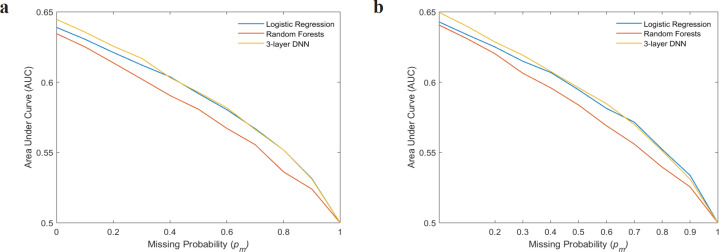
Shows Variation of Mean AUCs with Missing Probability, *p*_m_, for All the Three Classifiers on (a) validation set (b) test set

Figure 1a and 1b show the variation of mean AUCs with missing probability p_m_ for logistic regression, random forests and the NN on the cross-validation and model testing sets, respectively. As expected, the performance of all the classifiers were degraded with an increase in the percentage of missing information. Overall, the mean AUC was observed to be high for NN when compared to other classifiers up to p_m_ < 0.5. But when the percentage of missingness exceeded 50%, both logistic regression and NN performed equally well. The values of mean AUCs at p_m_ = 0 were slightly different from Table 2, as these values were obtained after retraining the classifiers by introducing additional 10% missing values during the first training.

*Performance of classifiers with inaccurate information*

**Figure 2:**
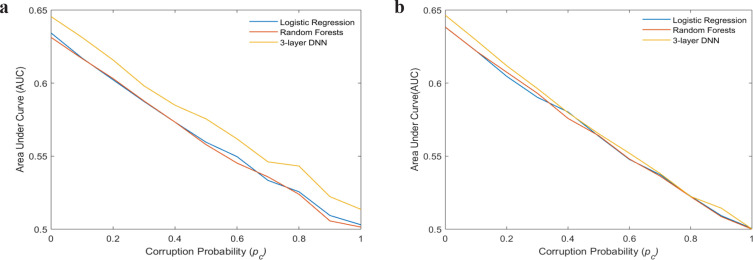
Shows Variation of Mean AUCs with Probability, *p*_c_, for All the Three Classifiers on (a) validation set (b) test set

Figure 2a and 2b show the variation of mean AUCs with corruption probability p_c_ for all the three classifiers, on the cross-validation and model testing sets, respectively. Similar to the above, the mean AUC reduced with the increased percentage of incorrect information for all the classifiers. NN resulted in superior performance in terms of AUC on the cross-validation sets, whereas on the independent test set, the performance was very close to logistic regression and random forests when more than 50% of the data was incorrect. It is important to note that the mean AUCs at p_c_ = 0 were slightly different from the mean AUC’s at p_m_ = 0 since these values were obtained by introducing the incorrect feature values with 0.1 corruption probability during training.

**Table 1:** Risk Factors and Coding Schema Used in the BCSC Dataset. Any category other than age that was not explicitly coded or was missing/unknown had been assigned a fixed code. (*) represents the total incidence for that category. The number of occurrences for each category is given in the parentheses

Risk Factor	Allowed values (number of occurrences)
Menopause status	Pre-menopausal: 0 (568,215)
	Post-menopausal or age ≥ 55: 1(1,642,824)
Age group (did not contain missing values)	35 to 39: 1 (42,758)
	40 to 44: 2 (287,281)
	45 to 49: 3 (387,246)
	50 to 54: 4 (428,312)
	55 to 59: 5 (334,132)
	60 to 64: 6 (263,521)
	65 to 69: 7 (231,904)
	70 to 74: 8 (203,106)
	75 to 79: 9 (145,102)
	80 to 84: 10 (69,636)
Breast density	Almost entirely fatty: 1 (148,209)
	Scattered fibro-glandular densities: 2 (782,384)
	Heterogeneously dense: 3 (674,008)
	Extremely dense: 4 (136,011)
Race	White: 1 (1,738,015)
	Asian / Pacific Islander: 2 (102,998)
	Black: 3 (121,534)
	Native American: 4 (28,359)
	Other or mixed: 5 (22,288)
Hispanic ethnicity	No: 0 (1,749,604)
	Yes: 1 (157,340)
Body mass index (BMI)	10.00 to 24.99: 1 (508,897)
	25.00 to 29.99: 2 (325,352)
	30.00 to 34.99: 3 (144,823)
	35.00 or greater: 4 (77,821)
Age at birth of first child	Under 30: 0 (722,195)
	30 or over: 1 (141,287)
	Nulliparous: 2 (201,222)
Number of first-degree relatives affected with breast cancer (NRBC)	None: 0 (1,718,360)
	One: 1 (295,768)
	Two or more: 2 (15,551)
Prior breast procedure performed	No: 0 (1,722,256)
	Yes: 1 (420,430)
Last mammography result	Negative: 0 (1,799,934)
	False Positive: 1 (34,046)
Surgical menopause	Natural only: 0 (717,966)
	Surgical: 1 (427,332)
	Not menopausal: 9 (568,215)
Ongoing Hormone Replacement Therapy	No: 0 (729,196)
	Yes: 1 (683,350)
	Not menopausal: 9 (568,215)

**Table 2:** Comparison of the Discrimination Metric in the Validation and Test Sets (mean AUC, and range of AUC derived from bootstrap sampling) Using Either the Native BCSC Dataset Encoding or (*) One Hot Encoding to the Data

	Validation mean AUC	Test mean AUC
Logistic Regression*	0.639 (range: 0.637-0.641)	0.645 (range: 0.644-0.645)
Random Forest	0.636 (range: 0.633-0.639)	0.643 (range: 0.642-0.643)
3- layer NN	**0.642 **(range: 0.638-0.650)	**0.649** (range: 0.647-0.651)

**Figure 3:**
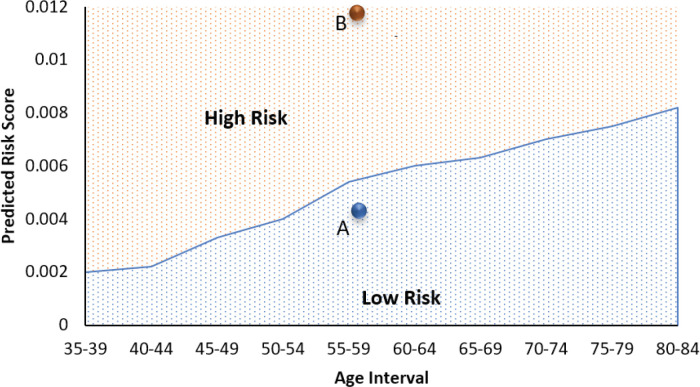
Illustration of Two Hypothetical Examples (Female A and Female B, as discussed in the text). For each example, the predicted risk of breast cancer within 1 year of screening is superimposed over the observed proportion of breast cancer diagnoses in the BCSC population as a function of age bracket

## Discussion

The primary aim of this work was to evaluate three commonly used classifiers in terms of their robustness towards missing and inaccurate data when predicting breast cancer incidence risk. The original BCSC dataset itself came with approximately 20% of missing data. NN showed superior results to logistic regression and random forests on this dataset as shown in Table 2. This superiority remained the same even when the percentage of missingness or incorrect information increased to 50%. When more than half the data was incorrect (p_c_ > 0.5) or missing ( p_m_ > 0.5), NN and logistic regression resulted in similar performance. The performance of RF remained low compared to other classifiers with varying missing data. 

Overall, the NN was observed to be a marginally superior choice for risk estimation of breast cancer from structured data like BCSC dataset, even when there was a large amount of missing and incorrect information. We also believe that these superior results with NN could be replicated on other health care datasets where there are high chances of incomplete or inaccurate information. In addition, the use of NN reduces the need for explicit feature engineering steps such as feature elimination, dimensionality reduction and missing values imputation, due to the capability of NN to learn non-linear transformations from inputs to outputs. To effectively train an NN to deal with incomplete and inaccurate information, future work could attempt to increase the probabilities of missing ( p_m_) and incorrect information (p_c_) during the training of NN instead of fixed ‘0.1’ as discussed in our experimentations. This may aid the NN to see the possible instances of missing or inaccurate information that might happen in the real setting.

To communicate the risk score so that it is more interpretable and explainable, we expressed this result as the relative risk compared to all women of the same age bracket. For example, Female A was a naturally menopausal 57-year old woman of Asian descent, with BMI under 25 and having her first child while under 30 years of age. She had no family history of breast cancer nor any prior breast surgical procedure. All her previous mammograms were negative, but her breast tissue density was not recorded (see Figure 3). was not recorded (see Figure 4). Our best model estimates that Female A has slightly less risk than the overall average risk of all women in her similar age group. In contrast, Female B was a nulliparous 57-year-old woman of Asian descent with a BMI of 35. She had dense breast tissue and two of her relatives died of breast cancer. However, none of her previous mammograms were positive and she had no previous breast surgical procedures. Our model illustrates that Female B has three times extra risk of breast cancer compared to women in this age group. 

We believe that the above-mentioned approaches could be used to help screening programmes to be more resource- and cost-effective by identifying high risk persons in an asymptomatic population. Individual risk-based screening can be extremely helpful when there is a constraint on the diagnostic resource usability, which is a frequently encountered scenario in developing countries. Risk estimation can direct these scarce diagnostic resources available for women who would need utmost, therefore it complements efforts to improve general access to screening. Also, the use of multiple risk factors can produce a better estimate of risk compared to traditional approaches (Deandrea et al., 2016) involving age alone as a risk criterion. We have currently hosted the risk estimation model proposed in this paper on our online website at https://www.niramai.org for public access. 

At this time, the wider clinical generalizability of this study is limited on a few fronts. First, we have not yet been able to independently validate the models using a more recent dataset or in the Indian population, due to a current lack of open access anonymized data. That is the focus of our future work and we further plan to validate our models in the Indian setting. Since the model is already trained to understand a diverse spread of risk factors from the BCSC data, convergence of a revised model in a new population setting is expected to be achieved much faster. Secondly, the combination of risk factors encompassing genetics, family history and individual phenotype is hypothesized to improve our ability to estimate breast cancer risk. Currently, there are no publicly available datasets with all the risk factors for the same population. A last observation would be that it is possible to either impute the missing values or detect potentially incorrect information by means of a conditional generative adversarial framework (Mirza and Osindero, 2014), but is outside the scope of the present study.

In conclusion, we compared three commonly used classes of machine learning classifiers - logistic regression, random forests and neural networks - for their robustness towards missing values and inaccurately reported values that are inherently present in population survey datasets such as the BCSC. The NN yielded marginally higher AUCs even when the data is missing or incorrect especially when their individual incidences are less than 50% for a breast cancer dataset. However, the performance of NN and logistic regression was equivalent when missing/inaccurate data was above 50%. Additional work is needed to validate the models in the Indian population, if these models are to be used to aid in selecting women for breast cancer screening. 

## Research Support

Siva Teja Kakileti and Geetha Manjunath are full-time employees of the private company Niramai Health Analytix. Co-authors Andre Dekker and Leonard Wee receive academic research funding from an Indo-Dutch Joint Research Programme grant BIONIC (no. 629.002.204) and a Dutch Technology Foundation research grant STW-Perspectief STRaTegy (no. 14930).

## Author Biography

Siva Teja Kakileti has 5 years of experience in the field of machine learning for Medical Imaging with over 15 publications and 6 US patents. He is currently one of the key data scientists at Niramai and is remotely pursuing PhD at Maastricht University.

Dr. Geetha Manjunath is the Founder, CEO and CTO of NIRAMAI. She has over 25 years of experience in IT research and has led many innovative projects in Healthcare and Transportation, especially catering to Emerging Market needs.

Prof. Andre Dekker, PhD (1974) is a medical physicist and professor of Clinical Data Science at Maastricht University in The Netherlands. He has authored over 150 publications in peer reviewed journals and holds multiple awarded patents.

Assistant Professor Leonard Wee PhD (1974) is a radio-oncology physicist specializing in medical imaging and machine-learning prediction models. He has authored papers, reviews and book chapters on radiomics, big data and outcome predictions that have been cited 400 times since 2015.
